# Gut-brain axis mediated by intestinal content microbiota was associated with Zhishi Daozhi decoction on constipation

**DOI:** 10.3389/fcimb.2025.1539277

**Published:** 2025-02-03

**Authors:** Leyao Fang, Xin Yi, Junxi Shen, Na Deng, Xinxin Peng

**Affiliations:** ^1^ The First Hospital of Hunan University of Chinese Medicine, Hunan University of Chinese Medicine, Changsha, China; ^2^ The Domestic First-class Discipline Construction Project of Chinese Medicine, Hunan University of Chinese Medicine, Changsha, China; ^3^ School of Traditional Chinese Medicine, Hunan University of Chinese Medicine, Changsha, China

**Keywords:** Zhishi Daozhi decoction, constipation, brain-gut axis, intestinal content microbiota, microbial diversity, high-fat and high-protein diet

## Abstract

**Background:**

Constipation is a common digestive system disorder, which is closely related to the intestinal flora. Zhishi Daozhi decoction (ZDD) is a traditional Chinese medicine prescription used to treat constipation caused by indigestion. This study is to evaluate the efficacy of ZDD in treating constipation and to elucidate the underlying mechanism.

**Methods:**

In this study, Kunming mice were administered a high-protein diet (HFHPD) and loperamide hydrochloride injections to induce constipation. The mice then received varying doses (2.4, 4.7, and 9.4 mg/kg) of ZDD for seven days. Following the sampling process, we measured fecal microbial activity. The levels of 5-hydroxytryptamine (5-HT), vasoactive intestinal peptide (VIP), and aquaporin-3 (AQP3) were quantified using enzyme-linked immunosorbent assay. Changes in the gut microbiota were evaluated through 16S rRNA gene sequencing. Additionally, we investigated the correlation between specific microbiota features and the levels of 5-HT, VIP, and AQP3.

**Results:**

The fecal surface of the mice in the model group (CMM) was rough and dry. The stool of mice in the low-dose ZDD group (CLD), medium-dose ZDD group (CMD), and high-dose ZDD group (CHD) exhibited a smoother texture, closely resembling that of the normal group (CNM). 5-HT levels in the CMM group were significantly lower than in the CNM, CLD, and CHD. VIP levels in the CMD were lower than in the other four groups, and AQP3 levels in CMM showed a decreasing trend. The fecal microbial activity of the CMM group was significantly higher than that of the other groups. Diversity analysis indicated that CMD and CHD treatments were more effective in restoring the intestinal microbiota structure. Potential pathogenic bacteria, including *Clostridium*, *Aerococcus*, *Jeotgalicoccus*, and *Staphylococcus* were enriched in CMM. In contrast, beneficial bacteria such as *Faecalibacterium*, *Bacillaceae*, and *Bacillus* were more prevalent in the CLD, CMD, and CHD. Correlation analysis revealed that *Streptococcus* and *Enterococcus* were positively correlated with VIP, while *Succinivibrio* showed a negative correlation with 5-HT.

**Conclusions:**

Constipation induced by HFHPD and loperamide hydrochloride disrupts the structure of the intestinal microbiota. ZDD appears to alleviate constipation, potentially through mechanisms linked to the brain-gut axis and its interaction with the intestinal microbiota. Among the treatment groups, the medium dose of ZDD demonstrated the most effective results.

## 1 Introduction

Constipation is characterized by difficult defecation, reduced bowel movement frequency, dry and hard stools, or a persistent sense of incomplete evacuation. It is a common gastrointestinal symptom linked to declines in both physical and mental health, significantly impacting overall quality of life (Black and Ford, 2018). Functional constipation affects up to 10% of children, with unhealthy eating habits being a common cause (Koppen et al., 2018; Taylor et al., 2016). A high-fat, high-protein diet (HFHPD) is frequently implicated in childhood constipation, as it may lead to dry stools and chronic constipation due to the difficulty in digesting fats (Liu et al., 2023). Researchers observed prolonged intestinal transit time and reduced colonic mucus production in mice subjected to HFHPD for eight weeks (Mukai et al., 2020). Additionally, mice treated with HFHPD for four weeks display a reduction in fecal volume (Shimada et al., 2021). In traditional Chinese medicine (TCM), this type of constipation is classified as gastrointestinal food stagnation syndrome, and it is associated with HFHPD (Qu et al., 2017). Loperamide hydrochloride, which increases stool consistency and firmness, is used to manage acute and chronic diarrhea. When combined with HFHPD, it successfully induces a constipation model in animals (Zhang et al., 2022).

The gut-brain axis is a bidirectional regulatory axis for the interaction between gastrointestinal function and the central nervous system (P. Chen et al., 2022). Gastrointestinal symptoms are commonly linked to mental disorders, such as depression and anxiety, which can worsen constipation (Liang et al., 2022). The intestinal microbiota take part in a number of physiological processes (Milani et al., 2017). It includes glycolysis, vitamin and metabolites production, and gastrointestinal immune responses, all of which are necessary for maintaining gastrointestinal homeostasis (Li et al., 2024; Wang et al., 2023). Recent research has established a connection between gut microbiota and constipation, highlighting how disruptions in the microbiota, impaired food decomposition, and reduced intestinal motility can contribute to constipation (Lai et al., 2023; Erhardt et al., 2023). Prolonged constipation can further alter the structure of the intestinal microbiota due to the accumulation of feces in the colon. The microbiota-gut-brain axis refers to the complex regulatory interaction between the intestinal microbiota and the gut-brain system (Shandilya et al., 2022). Key brain-gut peptides, such as 5-hydroxytryptamine (5-HT) and vasoactive intestinal peptide (VIP), are found in both the digestive tract and nervous system, facilitating gut-brain communication (Arzani et al., 2020). Additionally, 5-HT and VIP are gastrointestinal hormones that can indicate intestinal peristalsis (Olsson and Holmgren, 2001).

Several medications are available for treating constipation, but TCM offers a distinct approach. TCM treats gastrointestinal disorders with food stagnation by strengthening the spleen, regulating the stomach, and enhancing digestion to eliminate food stagnation. TCM’s multi-target, multi-channel approach has proven effective in alleviating this syndrome (Wang et al., 2022b). One key method in treating constipation is regulating the gut microbiota (Li et al., 2020). Zhishi Daozhi Decoction (ZDD), derived from the traditional formula Zhishi Daozhi Pills, is commonly used in TCM. Since decoctions act more rapidly than pills, they are preferred for research. ZDD consists of eight Chinese herbs. *Rheum officinale Baill.* can promote defecation by regulating the gut microbiota, stimulating colonic mucus production, and enhancing intestinal peristalsis (Gao et al., 2021; Ma et al., 2022). *Citrus aurantium* L. and *Atractylodes macrocephala* Koidz help regulate the intestinal microbiota and enhance gastrointestinal motility (Wang et al., 2022a). *Scutellaria baicalensis* Georgi and *Coptis chinensis* Franch. have the ability to control inflammation and lipid metabolism (Shen et al., 2024). Massa Medicata Fermentata modulates gastrointestinal hormone secretion and improves intestinal flora to support digestion (Jung et al., 2024; Fan et al., 2024). ZDD is used clinically to treat food stagnation in the gastrointestinal tract, as well as sticky and incomplete feces (Bi et al., 2022).

Previous research has demonstrated that ZDD alleviates constipation by modulating oxidative stress through its effects on intestinal mucosal flora (Peng et al., 2023). The impacts of the mucosal flora and the flora in the intestinal contents on the body are different (Zhang et al., 2021). The intestinal content flora regulates the production and release of braingut petide such as 5-HT and VIP (M. Chen et al., 2022). The intestinal flora can also affect the expression and function of aquaporins through its metabolites. This study investigates the mechanism of ZDD’s efficacy by examining key molecules involved in the brain-gut axis interaction and changes in the intestinal content microbiota.

## 2 Material and methods

### 2.1 Animals

To eliminate the influence of gender, we chose fifty SPF-grade male Kunming mice for this study (Wu et al., 2022). Mice were acquired from Hunan Silaike Jingda Experimental Animal Co., Ltd. Animal grouping. Animal Center of Hunan University of Chinese Medicine (License No: SYXK (Xiang) 2019-0009) maintains a temperature range of 23-25°C, relative humidity of 50%-70%, and a 12-hour light/dark cycle. This experiment was reviewed and approved by the Institutional Animal Care and Use Committee of Hunan University of Chinese Medicine., with ethics number: LLBH-202210260002.

### 2.2 Feed

Diet Preparation: A high-fat, high-protein diet was formulated using a mixture of milk powder (Nestle Nutritious Milk Powder), wheat flour (Huiyi Gluten Wheat Flour), floss powder (Zhenqiao Golden Floss), and soybean powder (Yonghe Soybean Milk Powder) in a 1:1:1:2 ratio (Zhou et al., 2022). Standard growth feed: The standard growth feed used in the study was sourced from the Animal Experiment Center at Hunan University of Chinese Medicine. Its nutrient composition included key indicators such as moisture, crude protein, crude fiber, crude fat, crude ash, calcium, total phosphorus, lysine, methionine, and cystine.

### 2.3 Medicine

Preparation of ZDD: ZDD was prepared using the following ingredients: 10 g of *Citrus aurantium* L. (Zhishi), 20 g of *Rheum officinale Baill*. (Dahuang), 6 g of *Coptis chinensis* Franch. (Huanglian), 6 g of *Scutellaria baicalensis* Georgi (Huangqin), 10 g of Massa Medicata Fermentata (Shenqu), 10 g of *Atractylodes macrocephala* Koidz. (Baizhu), 6 g of *Poria cocos* (Schw.) Wolf (Fuling), and 4 g of *Alisma orientate* (Sam.) Juzep (Zexie). All herbal ingredients were purchased from the First Affiliated Hospital of Hunan University of Chinese Medicine. The herbs were soaked in boiling water (10 times the combined weight of the ingredients) for 10 minutes and then filtered. Concentrate the filtrate in a rotary evaporator at 75 °C to create water decoctions with crude drug concentrations of 0.2 g/mL, 0.4 g/mL, and 0.8 g/mL based on mouse body surface area conversion.

Loperamide hydrochloride capsules: produced by Xian Janssen Pharmaceutical Co., Ltd. (Batch production No. MDJ7007).

### 2.4 Animal grouping and treatment

Fifty mice underwent a three-day period of adaptive feeding with free access to water and standard maintenance feed. Afterward, they were randomly divided into two groups: a normal control group (n=10) and a model group (n=40). The model mice were fed HFHPD. A dosage of 520 g/L milk, at 0.2 mL/10 g, was gavaged bi-daily for 14 days to simulate gastrointestinal food stagnation syndrome. Normal mice were fed general growth feed and given the same dose of sterile water as model mice via gavage for 14 days. Seven days after the HFHPD, the model group mice were intraperitoneally injected with loperamide hydrochloride at 0.3 g/ml once a day for seven days (Hajji et al., 2020). Normal mice were given normal saline intraperitoneally once a day for seven days. To assess the establishment of the constipation with gastrointestinal food stagnation model, we observe several key indicators, including fecal color and appearance, defecation time, and defecation frequency. Additionally, we monitor whether the mouse experiences incomplete defecation and evaluate its general activity and behavior. Successful model establishment is indicated by yellowish, sticky feces or hard stools that are difficult to expel, prolonged defecation time, reduced defecation frequency, incomplete defecation, and signs of digestive discomfort, such as loss of appetite and decreased activity (Deng et al., 2021; Peng et al., 2023).

**Figure 1:**
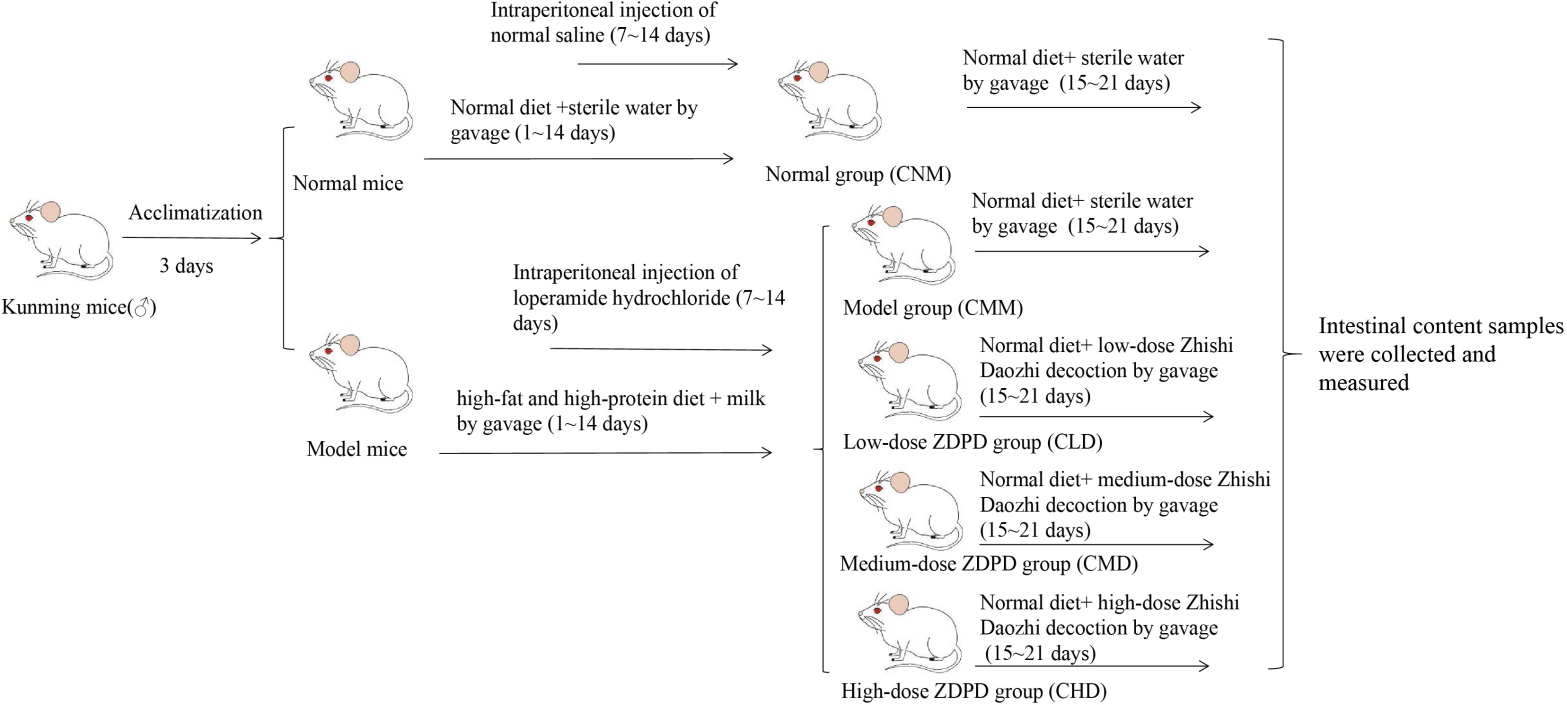
Experimental flow chart.

After successfully establishing the model, the normal mice were assigned to the normal group (CNM), while the model mice were randomly divided into four groups: model group (CMM), low-dose ZDD group (CLD), medium-dose ZDD group (CMD), and high-dose ZDD group (CHD), with ten mice in each group. Mice in the CLD, CMD, and CHD were administered ZDD at doses of 2.4 mg/kg, 4.7 mg/kg, and 9.4 mg/kg, respectively, via oral gavage. Each mouse received 0.35 mL at a time, twice daily, for seven days. The CNM and CMM groups were gavaged with an equivalent volume of sterile water under the same schedule (**Figure 1**).

### 2.5 General characteristics

During the experiment, the mice were observed and recorded from the aspects of fur color, mental state, activity, fecal characteristics, perianal cleanliness, weight, food intake, and water consumption at the same time every morning.

### 2.6 Fecal microbial activity

Fresh feces were collected by 1.5 mL sterile Eppendorf tubes from each mouse and stored at -20 °C after the intervention of ZDD. Pipette 2.5 mL of fluorescein diacetate (FDA) stock solution and add it to the sterilized phosphate buffer solution (solution A) with pH 7.6. Then, take a dry, sterile test tube, add 2 mL of solution A and 50 μL of the solution to be tested, put it in a shaker at 24 °C for 90 min, and then add 2 mL of acetone to stop the reaction. As a blank control, 50 μL of the solution to be tested and 2 mL of acetone were added to 2 mL of solution A in turn, and the mixture was taken out after shaking at 24 °C for 90 min. We measured the absorbance at 490 nm three times in parallel for each sample, and the A490 value expressed the microbial activity per unit mass sample (Zhou et al., 2022).

### 2.7 Determination of 5-HT, VIP and AQP3 contents

Whole blood samples were collected via ocular extraction and allowed to clot at room temperature for 2 hours. The samples were then centrifuged at 3000 r/min at 4°C for 10 minutes. The supernatant was collected for the analysis of 5-HT and VIP levels. The concentrations of 5-HT and VIP in the serum were measured using enzyme-linked immunosorbent assay (ELISA) kits, following the manufacturer’s protocols. After the mice were sacrificed on a sterile operating platform via cervical dislocation, the kidneys were extracted, and 0.1 g of kidney tissue was recovered. The kidney tissue was homogenized in an ice bath and centrifuged at high speed (4°C, 3000 r/min) for 10 minutes. ELISA was utilized to determine the kidney’s AQP3 level. The specific operations were performed in accordance with the kit instructions. The ELISA kits for VIP (SU-B20438), 5-HT (SU-B20715), and AQP3 (SU-B20477) were obtained from Quanzhou Kenuodi Biotechnology Co., Ltd.

### 2.8 Intestinal contents samples collection and 16S rRNA sequencing

Under sterile conditions, the contents of the jejunum to ileum segment were extracted using sterile forceps. The samples were individually placed into 1.5 mL sterile centrifuge tubes, labeled, weighed, and stored at -80°C in a freezer for intestinal contents microbiota analysis (Qiao et al., 2023). PCR amplification selected 16S rRNA V3-V4 region-specific primers of bacteria. The forward primer 338F (5’-barcode+ACTCCTACGGGAGGCAGCA-3’) and reverse primer 806R (5’-GGACTACHVGGGTWTCTAAT-3’) were used for PCR amplification of the 16S rRNA gene. Pre-denature the template DNA at 98°C for 30 s on PCR instrument, so that the template DNA is fully denatured, and then enter the amplification cycle. In each cycle, the template was denatured at 98°C for 15 seconds, and then the temperature was reduced to 50°C for 30 seconds to fully anneal the primer and template. Keep it at 72°C for 30 s, make the primer extend on the template, synthesize DNA, and complete a cycle. Repeat this cycle 25-27 times to accumulate a large number of amplified DNA fragments. Finally, the product was kept at 72°C for 5 min, so that the product was completely extended and stored at 4°C. The amplification results were subjected to 2% agarose gel electrophoresis, and the target fragments were cut out, and then the target fragments were recovered by the Axygen gel recovery kit. Then, 2×250 bp double-ended sequencing was performed on the Illumina NovaSeq machine using the Novaseq 6000 SP Reagent Kit (500 cycles). Sample DNA extraction, amplification, and library sequencing were completed by Shanghai Personalbio Technology Co., Ltd.

### 2.9 Bioinformatics

#### 2.9.1 Sequence processing and species annotation

The 16S rRNA sequencing was used to analyze the intestinal contents microbiota, and the modified and improved process was used to analyze the biological information of the microbiota. 100% sequence similarity is merged to generate characteristic sequence amplicon sequence variant (ASV). The ASV table is used to draw the species accumulation curve, which is used to detect the sequencing depth and evaluate the sequence data quality (Wang et al., 2018).

#### 2.9.2 Diversity analysis

The Chao1, Observed species, Shannon, and Simpson indices were calculated and analyzed using QIIME2 software. Principal co-ordinates analysis (PCoA) analysis was performed using the unweighted UniFrac distance.

#### 2.9.3 Characteristic microbiota analysis

The linear discriminant analysis effect size (LEfSe) method was used to detect the classification units with rich differences between the groups. To evaluate the diagnostic efficiency of the differential genera selected in the LEfSe analysis, a statistically significant receiver operating characteristic (ROC) curve was constructed for each differential genus, and the area under curve (AUC) was calculated (Fang et al., 2025).

#### 2.9.4 Correlation analysis

The Spearman correlation coefficient was used to analyze the relationships between characteristic bacteria and 5-HT, VIP, and AQP3.

#### 2.9.5 Metabolic pathway prediction

The metabolic functions of the microbial community were predicted by PICRUSt2 on the Kyoto Encyclopedia of Genes and Genomes (KEGG) database.

### 2.10 Statistical analysis

SPSS 25.00 software was used for statistical analysis, and the data of each group were expressed as mean ± standard deviation, and one-way ANOVA or Kruskal-Wallis test was used according to whether the data were normally distributed and the variance was consistent. *p* < 0.05 was considered a significant difference.

## 3 Results

### 3.1 General characteristics

**Figure 2:**
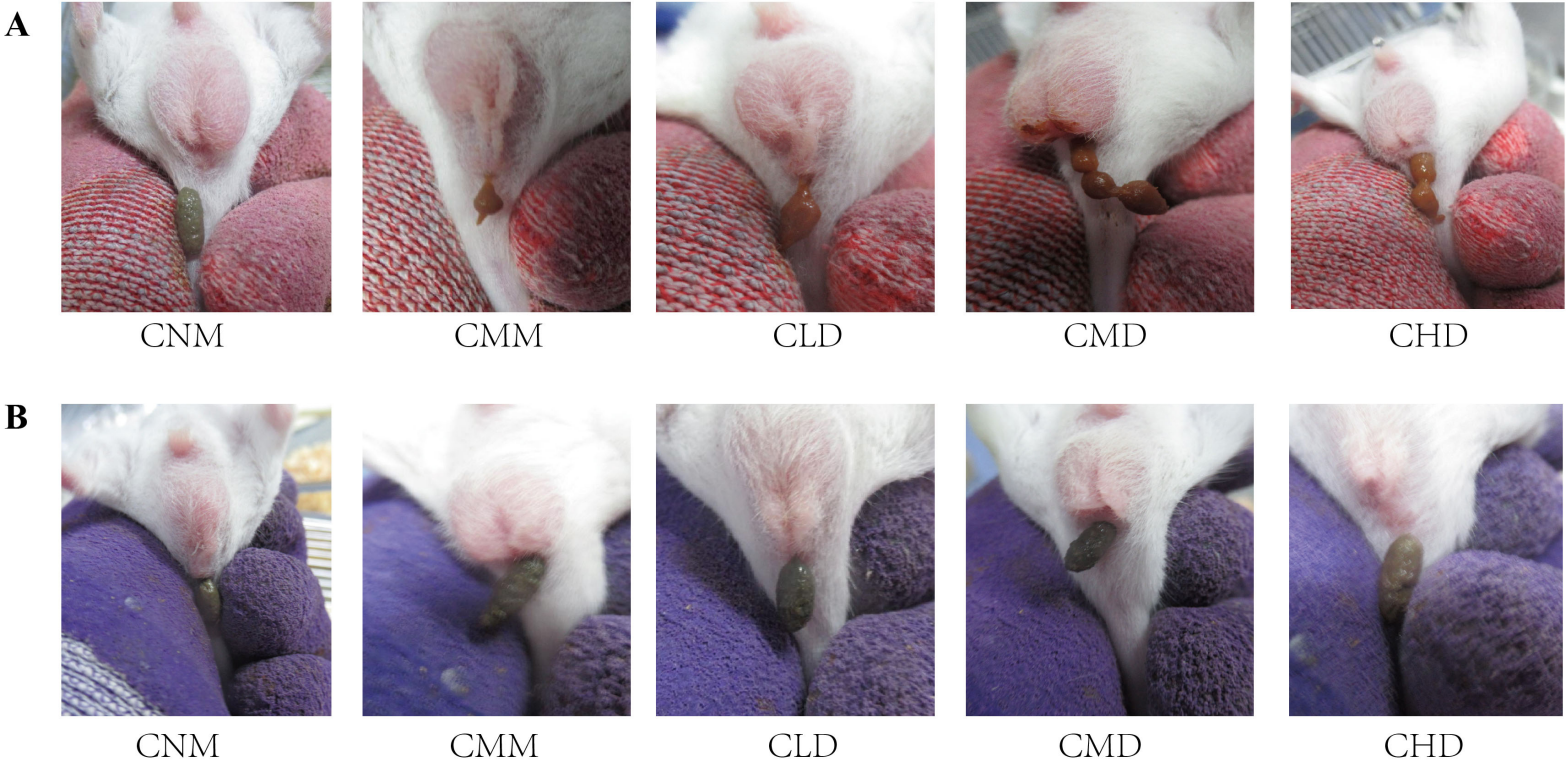
Fecal characteristics of mice. **(A)** Fecal characteristics of mice in each group after modeling. **(B)** Fecal characteristics of mice in each group after intervention with ZDD.

During the model establishment phase, mice in the CNM group displayed smooth fur, normal mental status, and dark brown stools with a well-formed shape and moderate hardness. In contrast, model mice exhibited signs of listlessness, reduced activity, huddling behavior, and yellowish, dull fur. Their food intake decreased, and their stools were sticky in texture, brownish-yellow in color, and had a beaded appearance. Additionally, defecation took longer and appeared incomplete, suggesting symptoms consistent with dyspepsia and gastrointestinal food stagnation (**Figure 2A**). The feces of the model mice showed stickiness and a yellow color, suggesting potential dyspepsia and food stagnation. The model mice also showed prolonged defecation time and incomplete bowel movements. During the ZDD intervention phase, the CMM group exhibited a poorer mental state compared to the CNM group, resulting in decreased autonomous activity. In contrast, mice in the ZDD intervention groups (CLD, CMD, and CHD) demonstrated improved engagement in independent activity. Additionally, fecal observations across all groups showed dark brown stools. The CMM group’s feces were rough, dry, and difficult in texture, while the feces from the ZDD-treated groups (CLD, CMD, CHD) were smoother in texture, more closely resembling those of the CNM group (**Figure 2B**).

### 3.2 Contents of 5-HT, VIP, and AQP3

**Figure 3:**
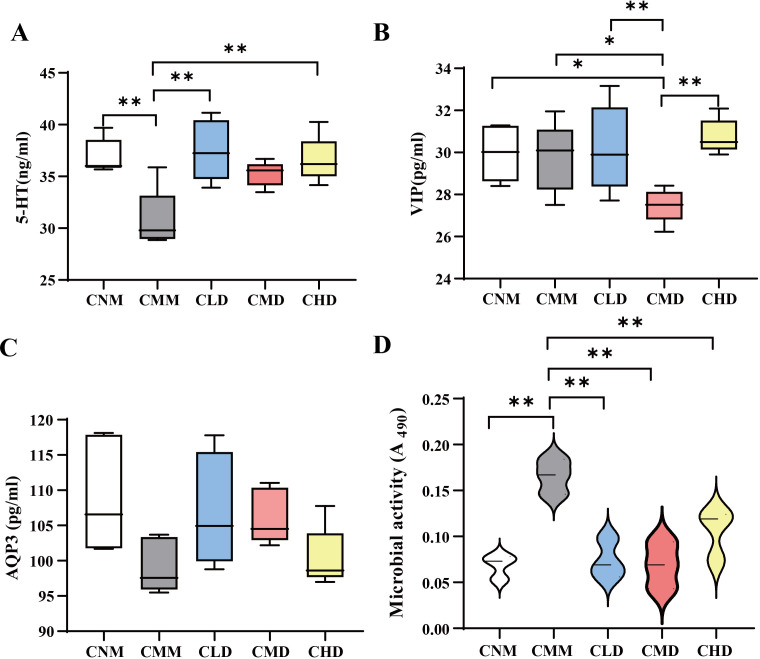
Contents of 5-HT, VIP, AQP3, and fecal microbial activity. **(A)** Contents of 5-HT. **(B)** Contents of VIP. **(C)** Contents of AQP3. **(D)** Fecal microbial activity of mice after the intervention of ZDD. Data are expressed as mean ± standard deviation. *p<0.05, **p<0.01.

5-HT plays a key role in stimulating intestinal smooth muscle contraction, promoting peristalsis, and accelerating colonic transit (Gershon, 2013; Israelyan et al., 2019). As shown in **Figure 3A**, 5-HT levels were significantly lower in the CMM group compared to the CNM group (*p* < 0.01), and were also reduced in the CMM group compared to the ZDD-treated groups. The 5-HT levels in the ZDD-treated mice were similar to those in the CNM group. **Figure 3B** illustrates that VIP levels in the CMD group were significantly lower than in the CMM group (*p* < 0.05) and also lower than in the CLD and CHD groups (*p* < 0.01). In **Figure 3C**, a decrease in AQP3 levels was observed in the CMM group. However, AQP3 levels in the CLD and CMD groups were approaching those observed in the CNM group.

### 3.3 Fecal microbial activity

The hydrolysis of FDA by nonspecific enzymes produced by bacteria and fungi results in fluorescein, with microbial activity being directly proportional to FDA hydrolase activity. This allows for a partial assessment of microbial metabolic capacity through the evaluation of intestinal microbial activity *in vitro* (Li et al., 2022). Consequently, we evaluated the fecal microbial activity of mice in each group following administration. The results indicated that the fecal microbial activity of CMM was significantly higher than that of CNM (*p* < 0.01), while the fecal microbial activity in CLD, CMD, and CHD was significantly lower than that of CMM (*p* < 0.01) (**Figure 3D**).

### 3.4 Assessment of the quality of intestinal contents microbiota sequencing data

**Figure 4:**
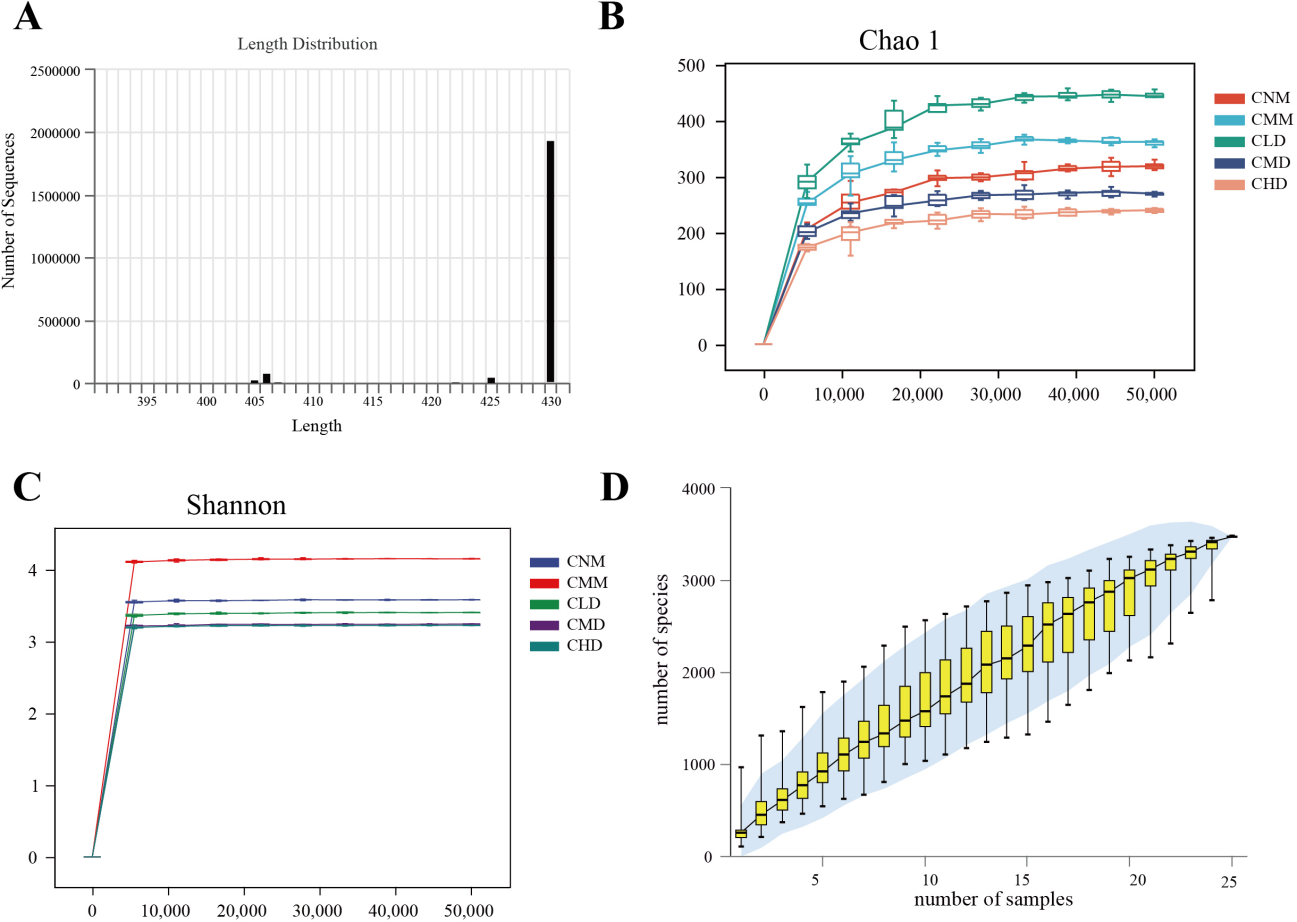
Quality assessment of intestinal content microbiota sequencing data. **(A)** Sequence length distribution diagram. **(B)** Chao 1 dilution curve. **(C)** Shannon dilution curve. **(D)** species accumulation curve.

The sequence length statistics derived from this sequencing indicate that the length distribution for each sample was predominantly concentrated between 400 and 440 bp (**Figure 4A**). The dilution curve shows that when the sequencing amount for each sample reaches 20,000, the curve enters a plateau phase, with the detected microorganisms in each sample approaching saturation (**Figures 4B, C**). The current sequencing depth adequately reflects the microbial diversity present in this sample batch. **Figure 4D** illustrates that as the sample increases, the rate of rise in ASV numbers diminishes, resulting in a flattening of the curve. This shows that, even with the addition of new samples, the total number of ASVs has only slightly increased, indicating that the samples in this study sufficiently meet the research objectives. We assume that this study uses an adequate sequencing depth, which ensures that the sequenced data accurately reflects the true composition of microbial communities in each sample, allowing for the analysis of microbial diversity in this batch of samples.

### 3.5 ASV numbers and diversity of intestinal contents microbiota in mice

**Figure 5:**
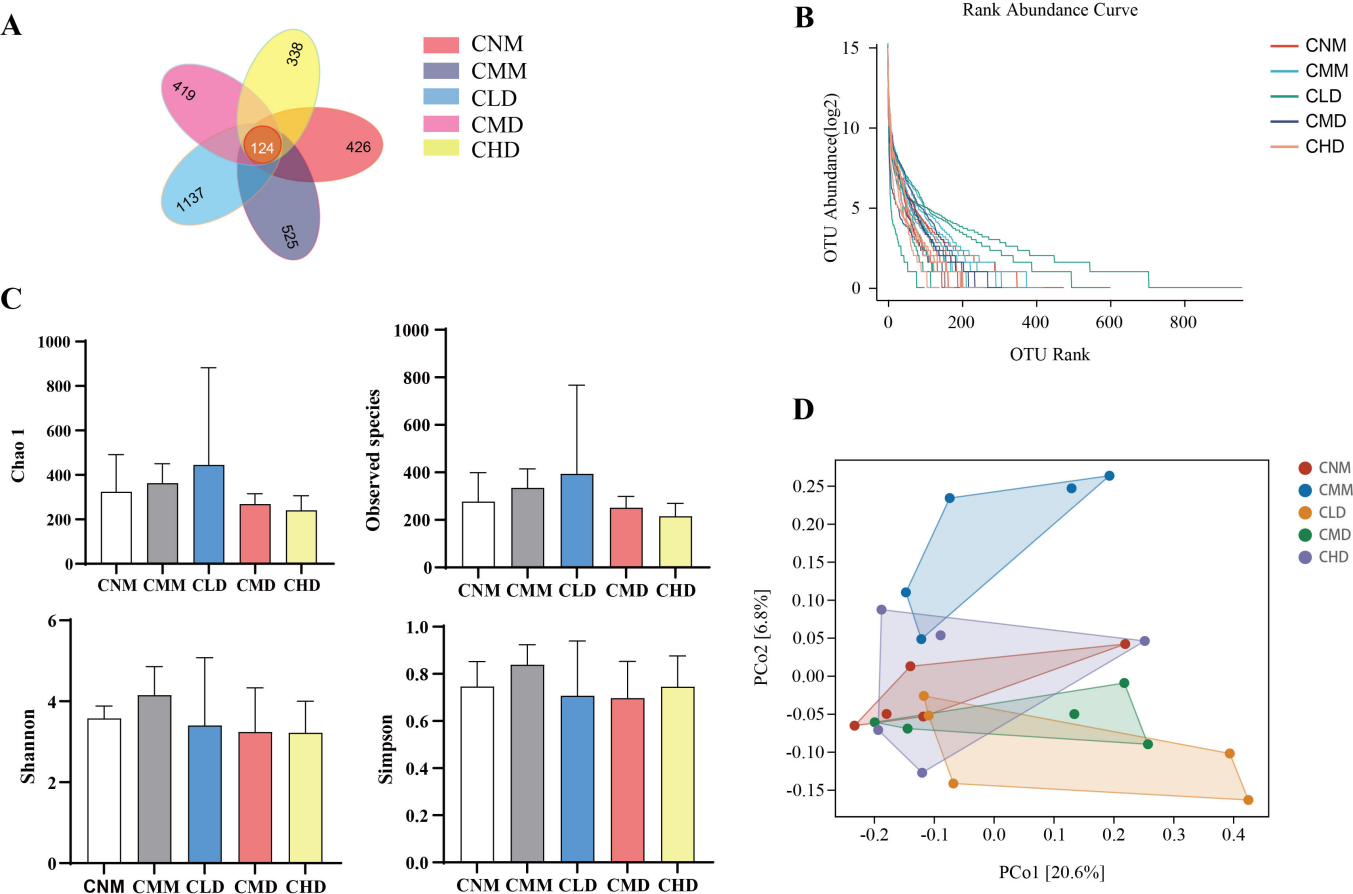
The number and diversity of ASV in the microbial community of intestinal contents. **(A)** Venn diagram. **(B)** Rank abundance distribution curve. **(C)** Alpha diversity index. **(D)** PCoA. Data are expressed as mean ± standard deviation.

The total number of ASVs for the CNM, CMM, CLD, CMD, and CHD were 426, 525, 1137, 419, and 338, respectively. The number of ASVs identified across the five groups is 124 (**Figure 5A**). The rank abundance distribution curve is constructed using the log2 values of abundance (**Figure 5B**). The highest number of ASVs was observed in CLD, aligning with the Venn diagram. To evaluate the alpha diversity of microbial communities, we utilize the Chao1 index and observed species index to assess richness, while the Shannon and Simpson indices are employed to characterize diversity (Gail et al., 2021). Chao 1 index and Observed species index in CLD showed an upward trend, which indicated that the intervention of low-dose ZDD regulated the intestinal microbiota richness to some extent, but the difference was not significant (*p* > 0.05). Shannon index and Simpson index in CMM showed an upward trend, indicating that constipation changed the diversity of intestinal microbiota to some extent (**Figure 5C**). Beta diversity describes the differences in species composition between communities in different habitats. PCoA demonstrated a distinct separation between the CMM and CNM, suggesting that constipation modifies the microbiota composition of intestinal contents (**Figure 5D**). Furthermore, there is no overlap between CMM and CLD, CMD.

### 3.6 Analysis of the dominant microbiota of intestinal contents in mice

**Figure 6:**
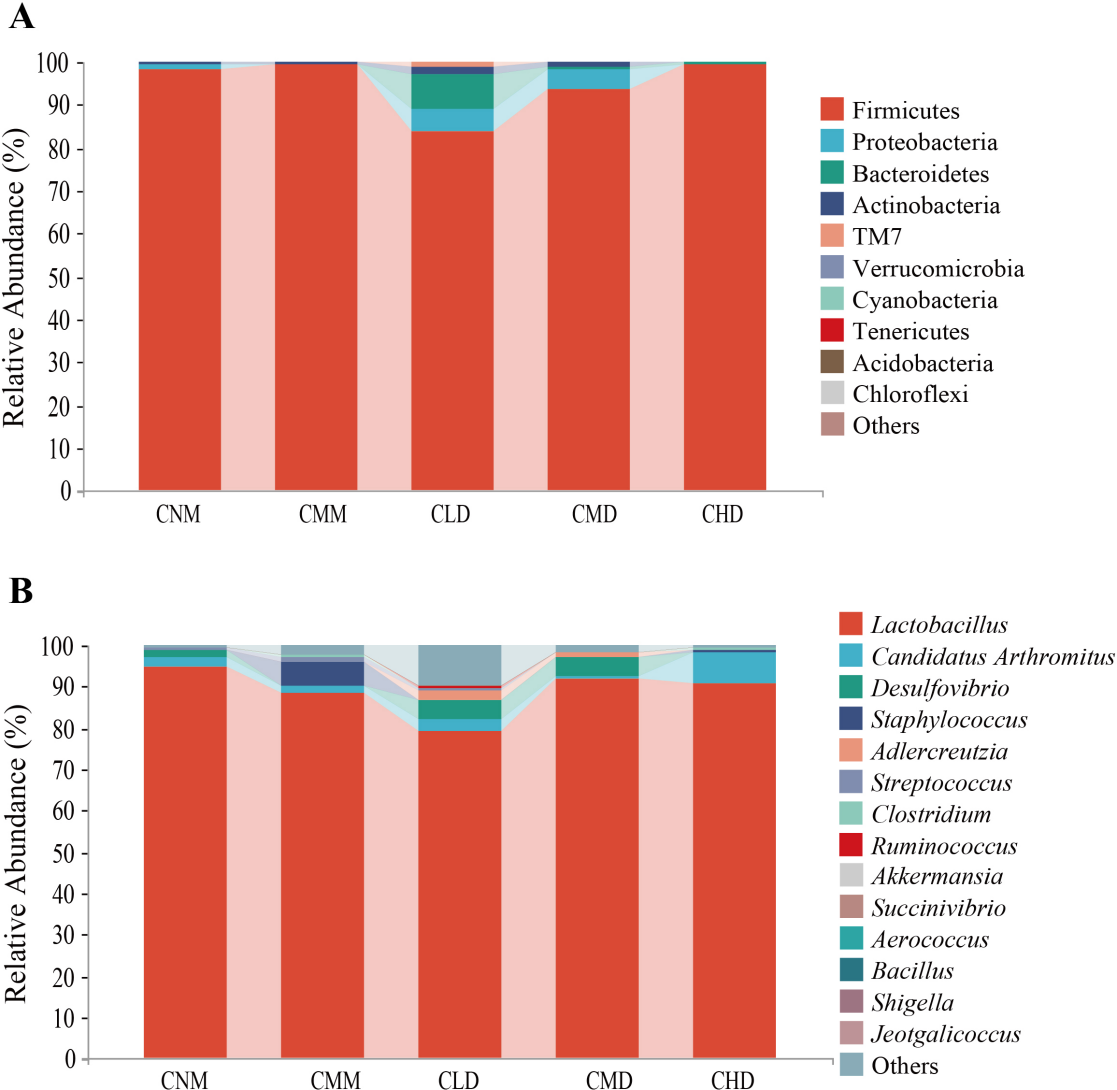
Dominant microbiota of intestinal contents. **(A)** The phylum level. **(B)** The genus level.

We screened the 10 phyla and 15 genera with the highest relative abundance and expressed them with a histogram. At the phylum level (**Figure 6A**), Firmicutes emerges as the predominant phylum across all groups, representing 98.03%, 99.32%, 84.09%, 93.51%, and 99.41% in CNM, CMM, CLD, CMD, and CHD, respectively. Proteobacteria constituted the second most significant group, representing 1.49%, 0.23%, 5.12%, 4.52%, and 0.08%, respectively. Bacteroidetes were predominantly found in CLD, comprising 7.71% of the total. In the genus level (**Figure 6B**), *Lactobacillus* is the predominant genus, comprising 94.91%, 88.29%, 79.71%, 91.80%, and 90.67% in CNM, CMM, CLD, CMD, and CHD, respectively. *Candidatus Arthromitus* accounted for 2.36%, 1.93%, 2.76%, 0.83%, and 7.51%. In addition, *Desulfovibrio* mainly exists in CNM, CLD, and CMD, accounting for 1.42%, 5.01%, and 4.47%, respectively. The relative abundance of *Staphylococcus* in CMM was 5.58%, significantly exceeding that observed in other groups.

### 3.7 Analysis of the characteristic bacteria of intestinal contents in mice

**Figure 7:**
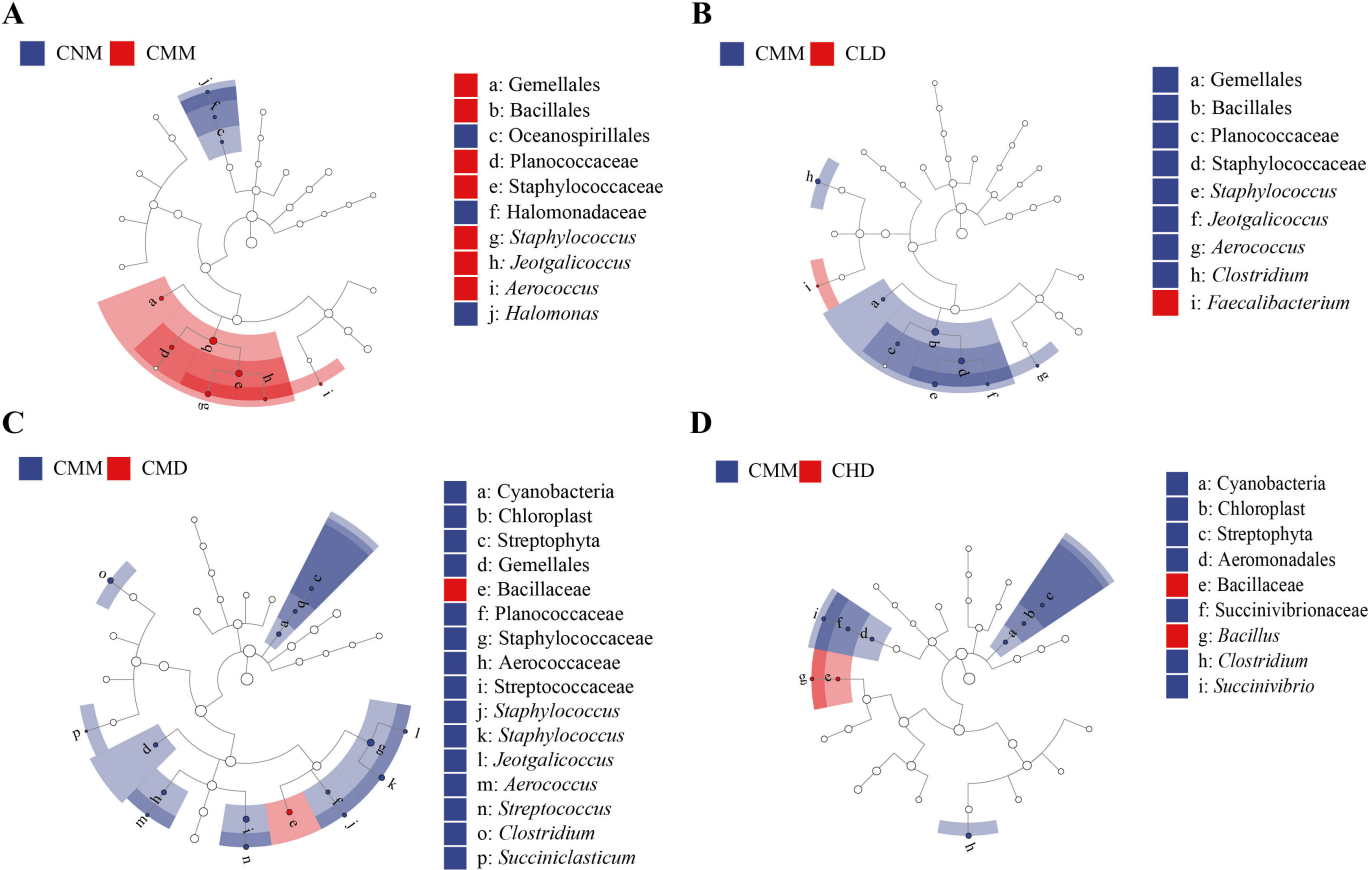
Characteristic bacteria of intestinal content between groups. **(A)** CNM and CMM. **(B)** CMM and CLD. **(C)** CMM and CMD. **(D)** CMM and CHD.

We used LEfSe with a logarithmic Linear Discriminant Analysis (LDA) threshold of 2.0 to find microbiota that differed significantly between groups. **Figure 7A** depicts the bacteria that distinguish CNM from CMM, with *Staphylococcus, Jeotgalicoccus*, and *Aerococcus* being particularly abundant in CMM. As shown in **Figure 7B**, *Staphylococcus*, *Jeotgalicoccus*, *Aerococcus*, and *Clostridium* are characteristic of CMM, whereas *Faecalibacterium* is diagnostic of CLD. *Bacillaceae* was the distinctive bacteria concentrated in CMD, whereas *Succiniclasticum*, *Clostridium*, *Streptococcus*, *Aerococcus*, *Jeotgalicoccus*, and *Staphylococcus* were characteristic bacteria in CMM (**Figure 7C**). In **Figure 7D**, the distinctive bacteria concentrated in CHD is *Bacillus*, while those in CMM are *Succiniclasticum*, *Clostridium*, *Streptococcus*, *Aerococcus*, *Jeotgalicoccus*, and *Staphylococcus*.

**Figure 8:**
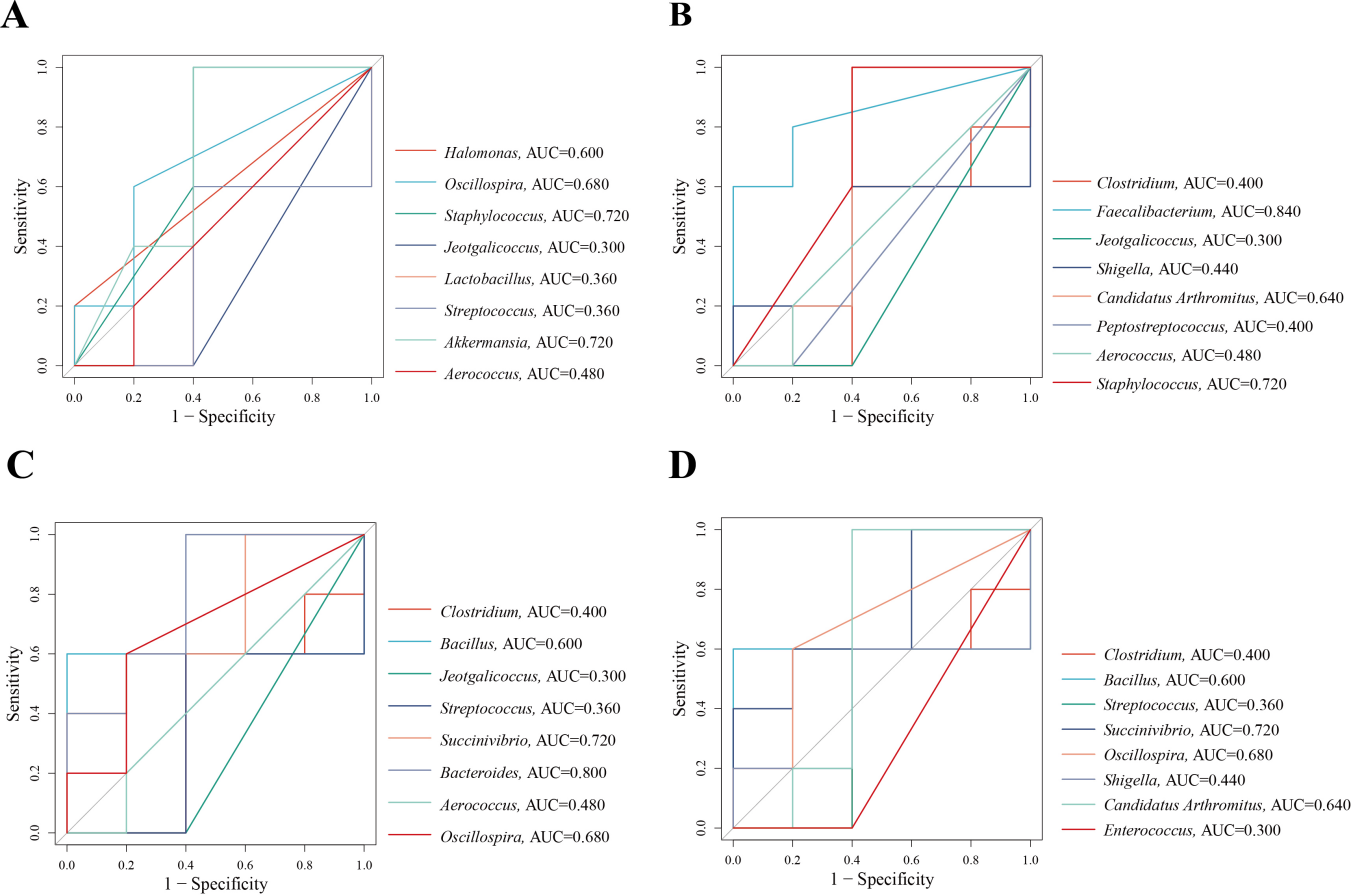
ROC curve. **(A)** ROC curve between CNM and CMM. **(B)** ROC curve between CMM and CLD. **(C)** ROC curve between CMM and CMD. **(D)** ROC curve between CMM and CHD.

The AUC represents the region bounded by the coordinate axis in a ROC curve. An AUC closer to 1 indicates a higher likelihood of distinguishing differences in microbiota composition between two groups in terms of relative abundance and diagnostic efficiency. We used an AUC threshold of > 0.8 to assess the diagnostic accuracy and identify distinctive bacteria across different groups. The ROC results suggest that the distinctive bacteria that contributed the most to the species level in CNM and CMM were *Staphylococcus* (AUC = 0.720) and *Akkermansia* (AUC = 0.720). In CMM and CLD, *Faecalibacterium* (AUC = 0.840) exhibited a high AUC. *Bacteroides* (AUC = 0.800) showed a high AUC in CMM and CMD. In CMM and CHD, *Succinivibrio* (AUC = 0.720) exhibited a high AUC value (**Figures 8A–D**). Characteristic bacteria with AUC > 0.8 are defined as important bacteria that describe the different characteristics between the two groups. These results indicated that *Faecalibacterium* and *Bacteroides* could be considered bacteria with diagnostic efficacy.

### 3.8 Correlation analysis between characteristic bacteria of intestinal content and 5-HT, VIP and AQP3

**Figure 9:**
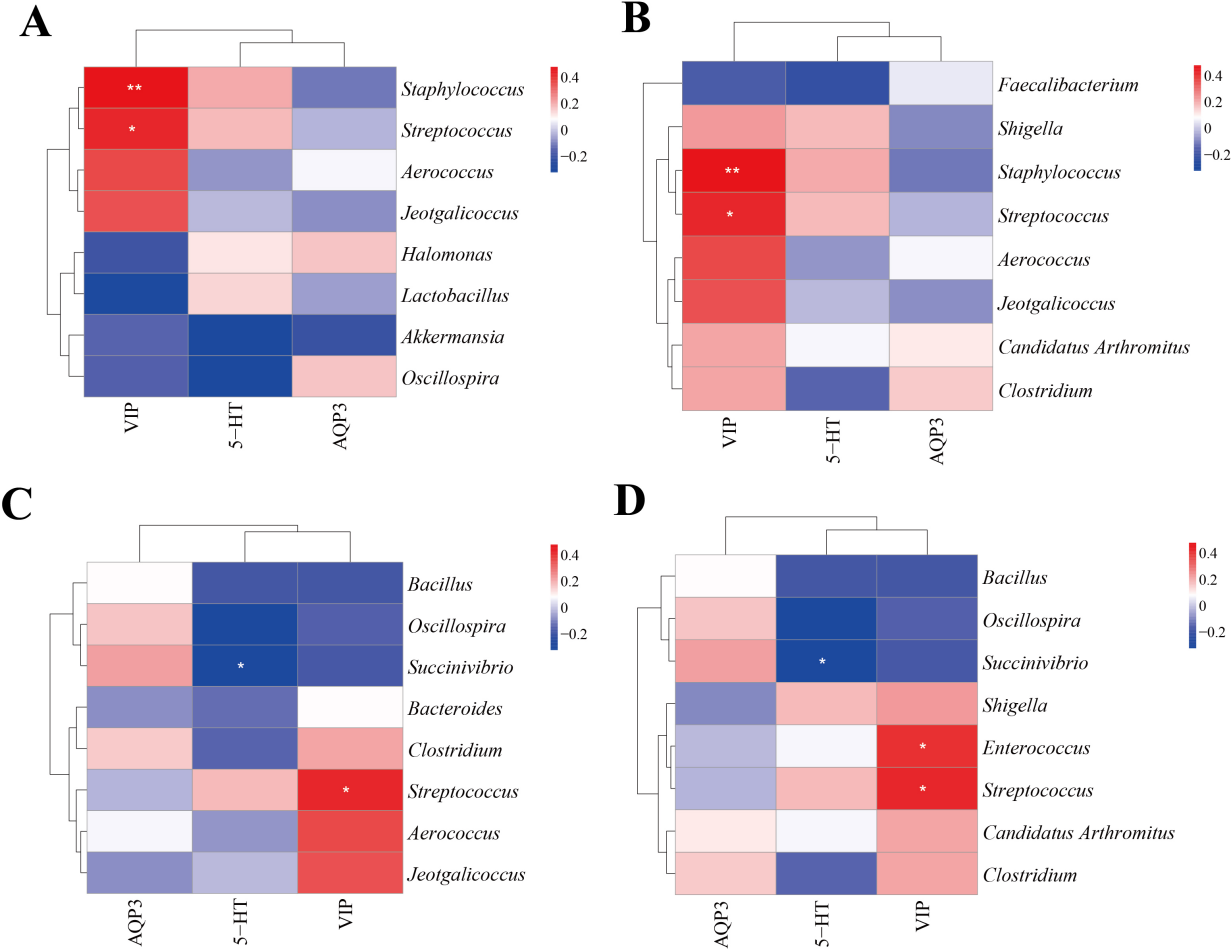
Correlation analysis. **(A)** Heat map of correlation between characteristic genus of CNM, CMM and each index. **(B)** Heat map of correlation between characteristic genus of CLD, CMM and each index. **(C)** Heat map of correlation between characteristic genus of CMD, CMM and each index. **(D)** Heat map of correlation between characteristic genus of CHD, CMM and each index. *p<0.05, **p<0.01.

We selected the characteristic bacteria with the highest abundance in the intestinal contents and conducted a correlation with 5-HT, VIP, and AQP3 indicators. **Figures 9A, B** illustrate the association between divergent microorganisms and markers in CNM and CMM, as well as between CMM and CLD. *Staphylococcus, Streptococcus*, and VIP had a notable positive connection (*p* < 0.01, *p* < 0.05). In the correlation analysis of distinctive bacteria and markers in CMM and CMD (**Figure 9C**), *Streptococcus* exhibited a positive connection with VIP (*p* < 0.05), but *Succinivibrio* had a negative association with 5-HT (*p* < 0.05). The study of the link between distinctive bacteria and markers in the CMM and CHD (**Figure 9D**) revealed a positive correlation for *Streptococcus*, *Enterococcus*, and VIP (*p* < 0.05), whereas *Succinivibrio* exhibited a strong negative correlation with 5-HT (*p* < 0.05).

### 3.9 Functional prediction analysis of intestinal contents microbiota in mice

**Figure 10:**
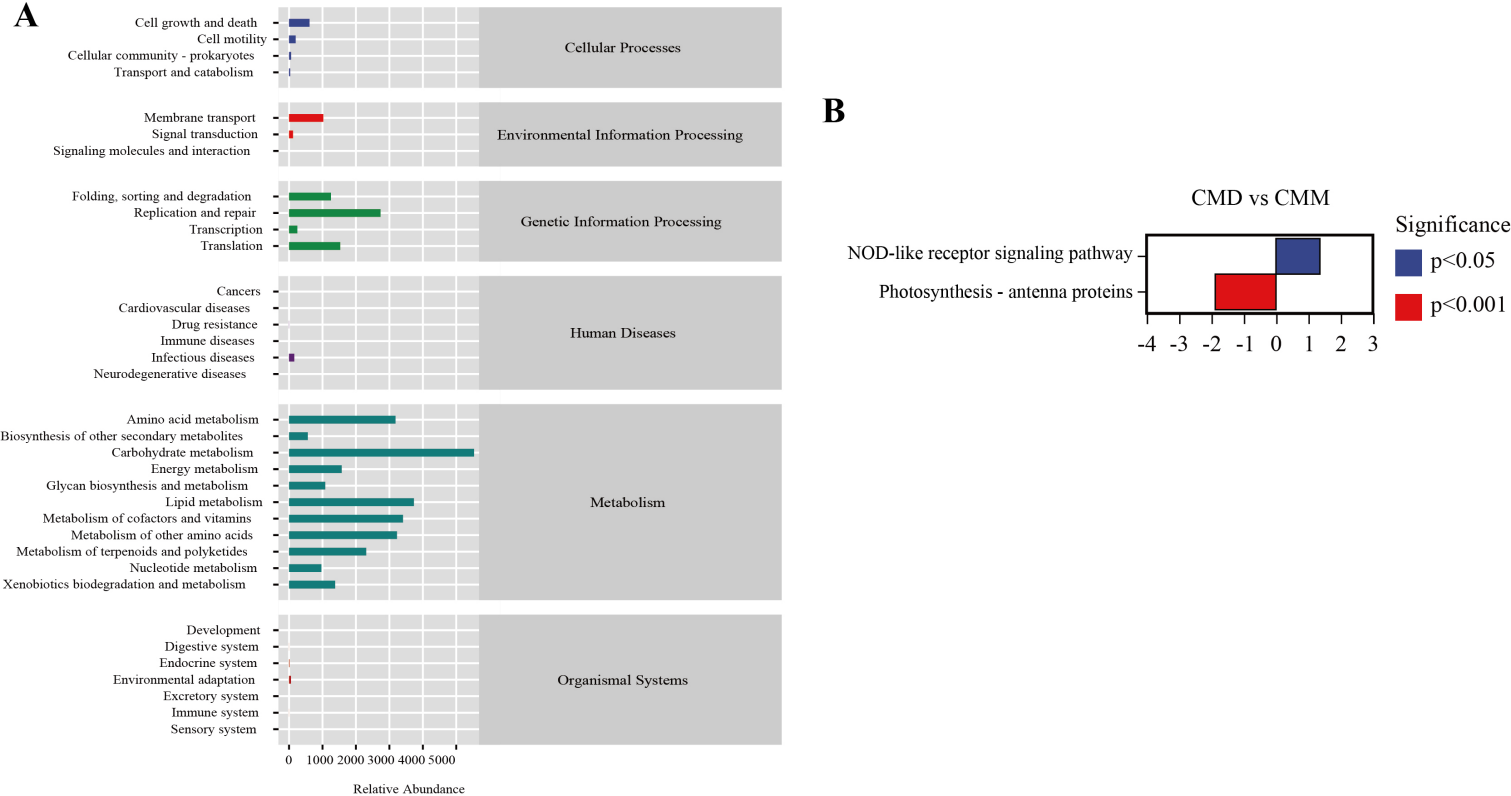
Functional prediction analysis of intestinal content microbiota. **(A)** Metabolic histogram. **(B)** Diagram of difference in metabolic pathways between CMD and CMM. Up-regulated group vs control group, positive horizontal axis in the figure represents up-regulated group compared with control group, while negative value is down-regulated.

The alteration of intestinal microbiota is concomitant with functional changes, so we conducted a function prediction analysis. Using the PICRUSt2 tool with the KEGG database, we categorized the functional profiles of the intestinal microbiota into six major classes: Organismal Systems, Metabolism, Human Diseases, Genetic Information Processing, Environmental Information Processing, and Cellular Processes. The secondary functional pathways comprised 35 subfunctions (**Figure 10A**). Among them, the metabolic pathways of abundance proofreading included Carbohydrate metabolism, Lipid metabolism, Metabolism of other amino acids, metabolism of cofactors and vitamins, Aminoacid metabolism, Replication and repair, and so on. We further analyzed the differences of metabolic pathways among the groups. Only CMD and CMM demonstrated changes in metabolic pathways. In CMD and CMM (**Figure 10B**), the nod-like receptor signaling pathway in CMD was dramatically up-regulated, whereas photosynthesis-antenna proteins were significantly down-regulated. Previous studies have emphasized the role of NOD-like receptors in gastrointestinal inflammatory diseases (Zhou et al., 2023).

## 4 Discussion

The gut and brain communicate through various pathways, including the enteric nervous system, vagal nerves, microorganisms, and their metabolites. Gastrointestinal symptoms such as constipation, diarrhea, and fecal incontinence are commonly observed in neurological disorders (Camilleri, 2021). Moreover, chronic constipation is often associated with cognitive decline (Ma et al., 2023). ZDD has been shown to alleviate constipation induced by HFHPD, which is linked to both constipation and negative impacts on brain health (Berding et al., 2021; Mukai et al., 2020). Intermittent fasting, by preventing overnutrition, can improve neurophysiological processes and reduce pathological damage (Guo et al., 2023). Treatments targeting the gut-brain axis have been found to relieve functional constipation (Singh et al., 2022). Modulating the gut-brain axis may be the underlying mechanism by which ZDD exerts its therapeutic effects. The model mice displayed reduced activity and decreased food intake, with stools that were sticky, brownish-yellow in color, and characterized by prolonged and incomplete defecation. These observations indicate that the constipation mouse model was successfully established. ZDD, at varying doses, was effective in alleviating constipation, with the medium dose showing the most pronounced effect. Additionally, ZDD improved the mental condition of the mice and increased their food intake. The 5-HT levels in the ZDD intervention group were similar to those in the CNM group, suggesting that ZDD may help restore gastrointestinal motility in constipated mice. VIP, which plays a role in relaxing smooth muscles, inhibiting gastrointestinal emptying, and reducing neural excitability (Bai et al., 2024; Seo et al., 2019), was found to be reduced in the CMD group. Changes in AQP3 levels can affect water absorption, influencing intestinal water metabolism and contributing to constipation (Ikarashi et al., 2016; Su et al., 2020). After ZDD treatment, AQP3 levels increased. Gastrointestinal motility was significantly impaired in the CMM group compared to the CNM group. These results suggest that ZDD effectively enhances gastrointestinal peristalsis and regulates water and fluid metabolism, leading to an improvement in constipation.

In our study, microbial activity in the feces of the CMM group was significantly higher. We hypothesize that constipation may promote the growth of certain bacteria or the expression of their functional genes. However, following the use of ZDD, microbial activity reverted to normal. Alpha and beta diversity analyses showed that constipation modeling altered gut microbiota richness, diversity, and structure. However, medium and high doses of ZDD progressively restored these parameters to normal levels. Further analysis at the genus level revealed a decrease in *Lactobacillus* abundance in both CMM and CLD groups, while the relative abundance in CMD and CHD approached that of CNM. *Lactobacillus* is a beneficial bacterium that produces short-chain fatty acids, which help promote colonic peristalsis (Kubota et al., 2020; Zhao et al., 2018). In addition, *Adlercreutzia* showed an increasing trend in the CMD group and is capable of synthesizing short-chain fatty acids (Sultana et al., 2022; Wei et al., 2018). LEfSe analysis identified several characteristic bacteria in the CMM group. *Succiniclasticum*, involved in succinic acid metabolism, may influence neuronal activity, with the gut microbiome potentially affecting brain function and behavior via succinate signaling (Zhang et al., 2024). *Clostridium* is linked to various human diseases, and an increase in its abundance has been associated with the progression of Alzheimer’s disease (Verhaar et al., 2021). The relative abundance of *Streptococcus* was significantly higher in elderly women consuming a high-protein diet, which aligns with our findings in the CMM group (Ford et al., 2020). *Staphylococcus* has been shown to impair immune function and contribute to chronic diseases (Ge and Sun, 2014; Paharik and Horswill, 2016). *Jeotgalicoccus* is related to lipid and carbohydrate metabolism (Li et al., 2021). *Aerococcus* is a common pathogenic or conditional pathogen, with its enrichment observed in patients experiencing functional constipation (Huang et al., 2018; Rasmussen, 2016). CMM showed a significant abundance of pathogenic bacteria, which may play a key role in the development of constipation. Following ZDD treatment, characteristic bacteria were identified, including *Faecalibacterium*, a major butyrate producer with anti-inflammatory properties (Eicher and Mohajeri, 2022; Faden, 2022), whose reduction is linked to depression (Valles-Colomer et al., 2019)*. Bacillaceae* is commonly used as a probiotic to alleviate symptoms of chronic intestinal disorders (Plaza-Díaz et al., 2017)*. Bacillus* is also considered important for alleviating constipation (Yang et al., 2024). In the ROC curve analysis, *Faecalibacterium* and *Bacteroides* showed an AUC greater than 0.8, indicating their diagnostic efficacy. Reduced *Bacteroides* in functional constipation patients may be linked to decreased gut motility and secretory function (Mancabelli et al., 2017).

Gut microbes communicate with the central nervous system through neuronal, immune, and endocrine pathways, influencing brain function and behavior (Liu et al., 2022). CMM exhibited characteristic bacteria associated with constipation, some of which are involved in gut-brain axis communication. We explored the correlation between 5-HT, VIP, AQP3, and these bacteria. *Streptococcus* and *Enterococcus* were positively correlated with VIP, while *Succinivibrio* showed a negative correlation with 5-HT. While the intestinal mucosal flora primarily interacts with the intestinal barrier and immune system, the flora within intestinal contents plays a critical role in digestion, nutrient absorption, metabolite production, and overall metabolic regulation. According to the correlation analysis between gut-brain peptide and intestinal microbiota, it is believed that there is a link between intestinal microbiota, 5-HT, and VIP. By regulating intestinal flora, the gut-brain axis can be modulated to enhance intestinal motility and secretion, benefiting constipation patients (Dimidi et al., 2017). The microbiota-gut-brain axis plays a key role in the pathogenesis of chronic constipation. ZDD can alleviate constipation by regulating the gut microbiota and influencing the production and release of gut-brain peptides.

## 5 Conclusion

Mice in the constipation model exhibited changes in brain-gut peptide levels. Pathogenic bacteria in the intestinal contents of constipation from gastrointestinal food stagnation syndrome are enriched, and the intestinal microbiota structure changes, which may be an essential factor in constipation. ZDD alleviates constipation by modulating the gut microbiota and influencing gut-brain axis communication. Additionally, our findings suggest that the medium dose of ZDD has the most pronounced therapeutic effect.

## Data Availability

The datasets presented in this study can be found in online repositories. The names of the repository and accession number can be found below: https://www.ncbi.nlm.nih.gov/, PRJNA936609.
